# Influence of Maternal Milk on the Neonatal Intestinal Microbiome

**DOI:** 10.3390/nu12030823

**Published:** 2020-03-20

**Authors:** Kathyayini P. Gopalakrishna, Timothy W. Hand

**Affiliations:** R. K. Mellon Institute for Pediatric Research, UPMC Children’s Hospital of Pittsburgh, University of Pittsburgh, Pittsburgh, PA 15217, USA; kpg13@pitt.edu

**Keywords:** maternal milk, breast milk, secretory IgA, neonatal microbiome, lactoferrin, human milk oligosaccharides

## Abstract

The intestinal microbiome plays an important role in maintaining health throughout life. The microbiota develops progressively after birth and is influenced by many factors, including the mode of delivery, antibiotics, and diet. Maternal milk is critically important to the development of the neonatal intestinal microbiota. Different bioactive components of milk, such as human milk oligosaccharides, lactoferrin, and secretory immunoglobulins, modify the composition of the neonatal microbiota. In this article, we review the role of each of these maternal milk-derived bioactive factors on the microbiota and how this modulation of intestinal bacteria shapes health, and disease.

## 1. Introduction

Mammals feed their infants milk for the period before they are capable of acquiring food on their own. Milk is also a mechanism by which a mother can protect the mucosal surfaces of their immunologically inexperienced infants from infection. In addition, maternal milk shapes the acquisition and development of the bacteria, archaea, viruses, protists, and fungi, collectively termed the microbiome, that colonize our barrier surfaces. The composition of the microbiome is tailored to particular tissue sites and functions [1]. While the initial colonization of the infant gastrointestinal (GI) tract with the microbiome can be volatile, there is a characteristic path for the development of the nascent microbiome after birth that is shaped by components of maternal milk. While breast-feeding likely has effects on microbial communities outside the GI tract, the most obvious and deeply studied effects are on the intestinal microbiota. Here, we will review the nutritional and bioactive components of milk and their potential effects on the developing microbiome of the infant intestine. 

## 2. Nutritional Components of Milk

The World Health Organization recommends exclusive breastfeeding to infants, as it provides all the nutrients required for the growth of the infant for the first 6 months of life and breastfeeding with food supplementation up to 2 years of age [2]. The nutritional components of human milk can be categorized into macronutrients (Table 1) and micronutrients (vitamins and minerals). Studies have observed that human milk changes in nutrition, composition, and quantity over time, with respect to different gestational periods (pre term vs. term), the diet of the mother, sex of the infant, and other environmental factors [3,4]. Human milk in the first few days of life is called colostrum, which is high in protein, whereas fats and carbohydrates are at lower concentrations. The importance of colostrum for the health of the newborn infant is illustrated by increased mortality rates in piglets who do not receive colostrum in the days directly post-delivery [5,6]. Maternal milk proteins can be classified into two main groups: antimicrobial/immune-stimulatory and nutritional. The proteins in the nutritional group assist in the absorption of vitamins and micronutrients in the intestine of the neonate and are a source of amino acids for the developing infant [7]. Colostrum, in particular, contains high concentrations of proteins that provide protection for neonates, including antibodies and other antimicrobials [3]. The anti-microbial proteins, listed in Table 2**,** will be discussed at length. Within each feed, the nutritional composition of milk also varies. For example, the initial milk or foremilk, contains large amounts of water and carbohydrates, whereas the milk that comes later, called the hind milk, is richer in lipids and proteins [8]. 

The fat concentration in human milk is highly variable and mainly consists of high concentrations of triglycerides of oleic and palmitic acids. The concentration of these lipids is influenced by the diet, especially the fat intake of the mother, thus explaining variation in the lipid concentration of the milk [3,10]. How fat and in particular milk-derived fat, directly affects intestinal bacteria is not well understood.

Lactose is the main source of nutritive carbohydrates in maternal milk and can be used for energy by some bacteria, particularly *Bifidobacteria* spp. and *Lactobacillus* spp. [11]. However, lactose is unlikely to be a significant driver of microbiome heterogeneity in infants because it does not reach the terminal ileum and colon where most intestinal bacteria live and its concentration is relatively conserved between different mothers [11]. Other carbohydrates secreted by the mammary gland include milk oligosaccharides (HMOs), [9]. Though non-nutritive to the infant, HMOs are digested by intestinal bacteria and their effects on the microbiome will be discussed below in detail [12,13].

The micronutrients in the human milk include vitamins A, B_1_, B_2_, B_6_, B_12_, and D, non-protein nitrogen-containing compounds and minerals, such as sodium, potassium, magnesium, and zinc [9]. The concentrations of these micronutrients vary significantly with availability of these components in the mother, which is highly dependent upon the diet. Many of these minerals are also requirements for various bacterial members of the microbiota. For example, zinc is a limiting reagent for the proliferation of multiple types of intestinal bacteria [14].

While all of these nutritional components are directly taken up by the host (except for HMOs), many of them will also affect members of the microbiota, either directly or via effects on host mucosal health. Malnutrition (both over and under delivery of nutrients) can have enormous effects on both the pediatric and adult microbiome [15,16,17,18], so it follows that if milk is enriched or deficient in a particular nutrient it could have important effects on the nascent infant microbiome. Further research will be necessary to determine how the different concentrations of the nutritive components of maternal milk shape the microbiome.

## 3. The Intestinal Microbiome

The intestinal microbiome is comprised of various bacteria, viruses, archaea, and fungi. Functionally, the intestinal microbiota provides the host with the enzymes necessary to digest complex carbohydrates and is therefore necessary for the enzymatic function of the intestine [19,20]. Specifically, the microbiome is required for the production of short chain fatty acids (SCFAs) from dietary fiber, which are then absorbed in the intestine both as an energy source and to modulate the immune system [21,22,23]. In adults, SCFAs (acetate, propionate, and butyrate) are largely produced by strict anaerobic bacteria, such as *Bacteroides* and *Firmicutes, that* together form a healthy and diverse microbiome. Thus, the presence of SCFAs is a biomarker of health of the microbiota [20]. Germ-free mice, which are bred to completely lack a microbiome, also have deficits in vitamin B and K synthesis, which can affect prothrombin levels [24]. Beyond this core digestive function, the microbiome is important to many other processes. For instance, many orally administered drugs are actually pro-drugs whose efficacy is dependent upon modification by the intestinal microbiota [25]. 

## 4. Development of the Intestinal Microbiota after Birth

The intestinal microbiota must develop from an initial state of low diversity, characterized by colonization with a limited set of microorganisms, dominated by facultative anaerobes (*Enterobacteriaceae*, *Enterococcaceae*) to a diverse and interconnected anaerobic community [26,27]. It takes about 3 years for a child to develop an intestinal microbiome composition similar to adults, i.e., dominated by *Bacteroides* and *Firmicutes* [26,27]. The infant stage of microbiome development is affected by factors such as prenatal exposure to antibiotics or toxins, the mode of delivery, antibiotic exposure after birth, diet (breast feeding versus formula feeding), the introduction of solid food, and environmental factors, such as geography or climate [26,27,28,29,30,31]. The colonization of the infant microbiome begins at delivery when infants can acquire many different types of bacteria from the environment and the vagina, skin, and intestine of their mothers [32]. It has been hypothesized that the early stage microbiome is also affected by a putative placental microbiota [33,34]. However, given the trace numbers of bacteria measured in placental studies, it is difficult to judge their importance in the face of colonization with millions of rapidly proliferating bacteria post-delivery. The microbial composition of infants changes substantially in the days directly post-delivery and is shaped by a variety of factors. Within the first hour of birth, infants delivered vaginally had mostly *Bacteroides* and *Lactobacillus* while infants born through caesarean section had increased levels of facultative anaerobes such as *Enterobacteriaceae*, *Enterococcaceae*, and other bacteria derived from the skin in their microbiota [32,35]. These differences, however, are short lived, and by one month, vaginal and C-section infants cannot be separated based upon the composition of their microbiota [36]. Infants exposed to antibiotics at birth also showed increases in facultative anaerobes, mainly *Enterobacteriaceae*, and a decrease in *Bacteroidetes*, *Bifidobacterium*, *Lactobacillus*, and *Clostridium* species as compared to unexposed infants [28,37,38]. Perhaps the most important modifier of the intestinal microbiome, regardless of the developmental stage, is diet. Accordingly, the diet of the infant has significant effects on the developing microbiota. Breast-milk-fed infants have an increased abundance of *Bifidobacterium* and *Bacteroides* while those fed formula maintain facultative anaerobes (*Enterobacteriaceae*) for longer periods of time [31,39,40]. The known bioactive factors (HMOs, antibodies, etc.) of maternal milk that contribute to these differences will be discussed below.

In cases where the acquisition of the microbiota is perturbed, a dysbiotic intestinal microbiota can colonize the infant and lead to diseases such as necrotizing enterocolitis (NEC) and bacterial sepsis [41,42,43]. Disruption of the microbiome early-in-life is also associated with a higher incidence of chronic disease, such as obesity and atopy/asthma, in animal models [44,45,46,47], and is widely hypothesized to predispose human disease. Indeed, since the intestine has evolved to carry a microbiota, it uses that microbiota for developmental cues and to direct various functionalities. Many of the important functions of the microbiota have been exposed by germ-free mice, which have immune related defects in effector CD4 T cell differentiation, macrophage-activated motility and intestinal epithelial regeneration [48,49,50,51]. Therefore, the shaping of the microbiota by maternal milk may affect lifelong health. Accordingly, the benefits of breast milk on development are well documented, and associations can be made between feeding infants a primary diet of formula and the development of acute and chronic disease [52,53,54]. For example, human milk reduces the incidence of NEC and sepsis in preterm infants, a disease that is almost certainly associated with shifts in the microbiome [55,56]. Breast-feeding also decreases the incidence of allergy, asthma and obesity [57]. The transfer of milk-derived immune factors from mother to child has direct effects on the development of the immune system, the prevention of neonatal infection (such as respiratory syncytial virus) and, by extension, chronic inflammatory disease [58]. However, many of these same factors have important roles in determining the composition of the microbiome and so determining mechanistic explanations for bioactive factors has been a challenge. Importantly, cessation of maternal milk feeding also has benefits for immune development. In mice, the microbiome of the weaning period is critical to the development of regulatory T cells that can dampen intestinal inflammatory responses later in life [59]. Interestingly, epidermal growth factor in maternal milk, which reduces disease in neonatal mice, prevents this weaning response [59,60]. Thus, while maternal milk establishes microbiome health, it must also cease to allow proper immune function into adulthood. Below, we will discuss the specific components of maternal milk that directly affect the microbiome and how this relates to disease. 

## 5. Human Milk Microbiota

Human milk is inhabited by its own microbiome that has been well characterized by culture and NextGen sequencing approaches [61]. While results have varied somewhat between studies, there has been a clear concordance where the milk microbiome is dominated by *Staphylococcaceae* and *Streptococcaceae*, with lesser amounts of *Lactobacilliaceae*, *Corynebacteriaceae*, and other organisms [61,62]. This structure is similar to the composition of the skin microbiome, and human milk is likely the source of the relatively large amounts of these bacteria that inhabit the neonatal intestine in the first month of life [63]. However, consistent breast feeding actually reduces the prevalence of these bacteria (in favor of *Bifidobacteria* and *Bacteroides*), so the importance of the milk microbiota in the development of the infant beyond the first few weeks of life is difficult to comprehend [31,39,40]. Further study is necessary to understand whether the human milk microbiome is necessary for establishing a healthy host/microbiome interaction in the infant. 

## 6. Bioactive Components in Developing the Microbiome

The bioactive factors of maternal milk are non-nutritional but have a significant role in preventing infections and maintaining the mucosal epithelium and the development of the microbiome. Bioactive factors primarily consist of anti-microbials, growth factors, and white blood cells, all produced by the mother and transferred to the neonate through milk. Their source can vary, as some are produced by the maternal mammary gland epithelium and others extracted from the maternal serum. The major bioactive factors are listed in Table 3. 

While, undoubtedly, many of these factors have direct functions in the development of the infant, here we will focus on those that are known to mediate their effects via modification of the microbiota.

## 7. Human Milk Oligosaccharides 

Human milk oligosaccharides (HMOs) are a group of glycans with a lactose backbone at the reducing end that can be elongated by linear or branching sugars (glucose, galactose, fucose, N-acetylglucosamine, N-acetylneuraminic acid, or sialic acid) varying in length from 3 to 15 carbohydrates [65,66]. The lactose backbone of the HMOs in the maternal milk can be further fucosylated, glycosylated, or sialylated, thus forming different structural isomers [66]. There is a high number of HMOs in the maternal milk, among which about 200 have been identified through mass-spectrometry [66]. HMOs are produced by the mammary gland and are present in large amounts in human milk, about 1g/dL [9,67]. They are not broken down by the digestive enzymes of the infant and thus can reach to the distal ileum and colon [68]. HMOs are variable in each mother depending on the genetic composition, diet, body weight of the mother during pregnancy, and the stages of lactation [69,70,71,72]. Differences in the concentration of HMOs have also been observed between mothers of preterm and term infants [73,74]. Variations in HMOs have been proposed to contribute to variability in the protective effects of milk from different women against neonatal disease [67]. For instance, glycosylated or fucosylated HMOs have a similar structure to the glycosylated moieties found on the surface of the intestinal epithelium [75]. In fact, prior to the identification of their structure, HMOs were thought to be glycoproteins or glycolipids secreted from the mammary epithelium [65]. The similarity of HMO glycosylated motifs to cell surface receptors allows them to interfere with the binding of certain bacteria to the epithelium [76]. Specifically, the presence of HMOs inhibits the growth of pathogenic bacteria like *Streptococcus pneumoniae* and *Campylobacter jejuni* by interfering with the adhesion of bacteria to epithelial cells [76,77,78]. HMOs have also been demonstrated to inhibit the growth of stable toxin-expressing *E. coli* by binding to the stable toxin [79]. Glycosylated HMOs also prevent NEC and rotavirus infection in experimental murine models [13,80,81,82].

*Bifidobacterium* and *Bacteroides* bacteria metabolize HMOs and utilize them as an energy source [83]. The digestion of HMOs by *Bifidobacteria* is especially beneficial for the infant since the by-product is often SCFAs [83]. Therefore, the provision of HMOs can be seen as a way to both foster a healthy microbiome while also delivering SCFA to the intestines of infants that lack the physical capability and microbiome to digest fiber. Since the only source of HMOs is milk, studies have shown that infants who are fed with mother’s milk have increased abundance of obligate anaerobes, such as *Bifidobacterium* and *Bacteroides*, in the first few weeks of life as compared to formula-fed infants [84,85]. The duration of breastfeeding influences the presence of *Bifidobacterium* in the infants. Infants from countries with a higher mean breastfeeding duration had about 80%–90% colonization by *Bifidobacterium longum* subsp. *infantis*, whereas infants from countries with a low mean breastfeeding duration had about 0.7%–14% *Bifidobacterium longum* subsp. *infantis* colonization [85]. Even though the presence of HMOs in the milk facilitates the growth of certain *Bifidobacterium* species, the ability to digest HMOs does not always coincide with the dominance of the different species [86]. The most dominant strains of *Bifidobacterium* in the infant stool are *Bifidobacterium longum* subsp. *longus* and *Bifidobacterium breve*, but these strains have a limited capacity to digest HMOs. In contrast, the strains with a high HMO digestion capacity, *Bifidobacterium bifidus* and *Bifidobacterium longum* subsp. *infantis*, are found in low abundance in the infant stool. The exact mechanism behind these differences is not clear because we still do not know the source of *Bifidobacterium* that colonizes the infant intestine, though it is clearly not the human milk microbiome (as discussed above). Colonization, HMO digestion and the growth of different *Bifidobacteria* spp. may be complex and involve interactions between multiple different organisms, and further research is warranted to better understand HMO digestion and its role in the microbiome of infants [86].

## 8. Lactoferrin and Other Anti-Bacterial Proteins Found in Milk

Lactoferrin, referred to as the “red protein from milk”, is an iron binding protein known for its bacteriostatic properties [87]. It is produced from the glandular epithelial cells of mammals and also secreted in granules from neutrophils [88]. It is a member of the transferrin family of proteins and strongly binds iron [88]. The amount of lactoferrin in the milk varies depending on the stage of lactation as colostrum contains a high concentration of lactoferrin and it slowly decreases through the first month of lactation [89,90]. The reduced digestive capacity of the infant intestine protects milk-derived lactoferrin from degradation and maintains its functional properties, thus restricting the growth of bacteria in early life [91].

Recognition of the iron binding ability of lactoferrin led to the discovery of its anti-microbial and immunomodulatory functions [92]. Lactoferrin’s best described function is the sequestration of iron by high affinity binding, thus making it unavailable to bacteria in the gut, which require iron as an essential component for growth [93]. This bactericidal action has been demonstrated for many bacteria, including *Streptococcus* spp. and *Vibrio* spp., but some strains of bacteria, in particular the more invasive/pathogenic strains, have evolved iron-binding mechanisms to counteract lactoferrin and obtain iron [93]. Outside of its iron sequestration function, lactoferrin is also believed to have other bacteriocidal and immunomodulatory effects. For instance, it prevents the formation of biofilms in the intestinal tract and the interaction of microbes with the host epithelial cells [92,94]. Lactoferrin also interacts with the lipopolysaccharide (LPS) of gram-negative organisms and inhibits their growth [95]. In some contexts, lactoferrin supplied in maternal milk may also have effects on the host immune response. Lactoferrin induces macrophage activation to aid in the phagocytosis of gram-positive bacteria [92]. Lactoferrin also increased the activity of Myeloid-Derived Suppressor Cells (MDSCs) in both infants and mouse models, thus preventing experimental necrotizing enterocolitis in mice [96,97]. Indeed, the supplementation of lactoferrin to enteral feeds may provide protection against late-onset sepsis and NEC in preterm infants [98].

In addition to lactoferrin, there are other anti-bacterial proteins found in maternal milk that may affect the development of the infant microbiome. Lactoperoxidase is an enzyme present in the tears, saliva, and milk of mammals. Lactoperoxidase catalyzes the oxidation of thiocyanate with hydrogen peroxide to hypothiocyanite. Hypothiocyanite exhibits its anti-bacterial property by decreasing the viability of bacteria, viruses, and fungi [99]. Haptocorrin is a Vitamin B12 binding protein, previously shown to inhibit the growth of bacteria, but more recent studies have shown that the bactericidal/bacteriostatic activity could not be attributed to haptocorrin alone, so perhaps haptocorrin functions in concert with other anti-bacterial mediators [100]. 

## 9. Secretory Immunoglobulins

One of the most important anti-microbial bioactive factors in the maternal milk are the immunoglobulins, which provide passive immunity from infections to the newborn infant. The main antibodies present in the maternal milk are IgA, IgM, and IgG. IgA constitutes about 90%–95% of all antibodies, with IgM accounting for 2%–5% and IgG is less than 1% [101]. IgG can cross the placenta via the neonatal Fc Receptor (FcRn) and provide protection to the fetus in-utero and the infant for months post-delivery. In some mammals, including rodents and ruminants, FcRn is also highly expressed on the duodenum and can mediate transport of IgG from milk [102]. IgA and IgM, however, cannot cross the placenta and thus are only provided to the neonate through maternal milk to facilitate protection against mucosal infection and shape the microbiome.

Of these three antibody subtypes, the role for IgA in establishing a healthy intestinal microbiota is most clear. IgA-deficient patients have a unique intestinal microbiota, characterized by an increased relative abundance of intestinal *Enterobacteriaceae* [103,104,105]. Similar data have been observed in mouse models lacking in IgA (Igha^−/−^ or Jh^−/−^ mice), as they had equal numbers of intestinal bacteria as IgA sufficient controls but their microbiota was dominated by *Enterobacteriaceae* and segmented filamentous bacteria [106,107,108].

The structure of IgA consists of two IgA monomers bound together by disulphide linkages between the constant region and the J-chain (joining chain), which is produced in B cells [109]. Dimeric IgA is transported across the epithelium by the polymeric Ig receptor (pIgR). During transcytosis, a portion of the polypeptide chain of pIgR gets covalently attached to IgA after cleavage at the luminal surface of the epithelium and is called the secretory component [110]. Thus, dimeric IgA after transcytosis across the epithelial layer is known as secretory IgA (sIgA). Secretory component prevents the proteolytic degradation of IgA and is partially responsible for the high IgA/IgM ratio in the intestine since IgM is transported by pIgR at the same rate but is not irreversibly bound and protected by secretory component [109,111].

Secretory IgA is produced in the mucosal surfaces and provides anti-bacterial effects by binding to bacteria and preventing them from invading the mucosal epithelium. Unlike IgG or IgM, the anti-bacterial activity of sIgA is not mediated by complement-driven cytolysis or through opsonization and phagocytosis because these functions are blocked by binding of secretory component. Instead, IgA-based immunity against bacteria is hypothesized to be mediated by a combination of factors including: a) steric hindrance of bacterial surface molecules, b) increased uptake or sampling by the M-cells in the Peyer’s patches, c) through modification of bacterial transcription, or d) “enchaining” bacteria to prevent gene transfer [112,113,114,115]. Not all of these mechanisms are equally relevant to sIgA derived from maternal milk. For example, the increased uptake of sIgA-bound bacteria by Peyer’s patches is unlikely to be important since pups of IgA-deficient mice show increased B-cell activation and antibody production due to the lack of maternal IgA [116]. Bacterial enchainment may allow for a more efficient removal of inflammatory/invasive bacterial isolates from the microbiome and additionally prevent them from interacting with other bacteria in the intestine and acquiring new genetic material [114]. Conversely, secretory IgA can actually support the colonization of certain bacteria, such as *Bacteroides,* by acting as a carbohydrate source [117]. It also supports the stable colonization of *Bacteroides* by a distinct mucosal niche through common colonizing factor (ccf) regulation, which enhances coating by sIgA and increases the epithelial adherence of those bacteria [118]. Together, this suggests that IgA regulates the growth of a healthy microbiome in the intestine by promoting the growth of obligate anaerobes, such as *Bacteroides* and *Firmicutes*, while limiting the growth of inflammatory facultative anaerobes, such as *Enterobacteriaceae*.

Studies in both mice and humans have shown that maternal milk is the only source of sIgA for pups in the first weeks of life [116,119,120]. This is likely because it takes about 3–4 weeks for the neonatal intestine to be populated by IgA-secreting B cells [120,121]. IgA in the milk is related to the maternal microbiome because during pregnancy, intestinal IgA+ B cells traffic to the mammary gland where they secrete IgA into milk, as directed by the chemokine CCL28 [122,123]. Recent studies in mice have identified the presence of B-cells with the same variable regions in both the intestine and mammary glands, confirming the enteromammary B cell circuit [124]. Thus, the sIgA secreted by mammary gland B cells is shaped by the intestinal microorganisms that drive the strongest intestinal IgA responses in the mother [125]. However, since the bacteria present in the infant are not necessarily shared with the mother, maternal IgA may not possess all of the specificities necessary to bind all of the bacteria found in an infant. This is particularly true in preterm infants whose microbiome is more shaped to their physical environment and therefore dominated by facultative anaerobes that are largely absent from the maternal intestine [126]. Additionally, bacterial invasion of the infant will not generally trigger a maternal response, and, therefore, escape from IgA binding due to mutation, horizontal gene transfer, or colonization with a new strain will proceed without modification of the dominant maternal IgA response. In accord with this hypothesis, the relative fraction of IgA-bound bacteria, specifically *Enterobacteriaceae,* decreases prior to the onset of NEC, even though these children are receiving human milk [120]. Therefore, the specificities of IgA present in breast milk might be as important as the amount of antibody in shaping the infant microbiota and that IgA binding is necessary to restrict the growth of *Enterobacteriaceae* in the intestine. Since the anti-bacterial IgA repertoire is dependent upon the intestinal B cell repertoire [124], it is likely to differ between mothers according to differences in the microbiome and infectious history. The protection provided by maternal IgA is potentially important to the development of a healthy relationship between the microbiota and the host immune system. In mouse models, a lack of maternal IgA has been associated with more rapid and more robust immune responses in pups. Specifically, the pups of IgA-deficient mothers produce their own IgA much more rapidly (prior to weaning) and also show increased T cell activation in gut-associated lymphoid tissue [116,127]. These differences in lymphocytes are potentially important to the long-term health of the infant due to the longevity of the cells, though more research on this subject is necessary. 

Despite its relatively low abundance in maternal milk, IgG has important effects on host-bacterial interaction in neonates. How IgG functions with regard to the microbiota is complex since it is transferred to the infant’s circulation (either *in utero* or via milk) or acts directly on bacteria within the intestine. However, it is clear that milk-derived IgG is critical for controlling enteric infections, independent of the effect of a placentally transferred antibody. *Enterobacteriaceae*-specific IgG in maternal milk has been shown to be critical for protecting neonatal mice against enteric infections with *Citrobacter rodentium* and enterotoxigenic *E. coli* [128,129]. IgG is also important for establishing homeostasis with regard to the newly colonizing microbiota by preventing the activation of the immune system in gut-associated lymphoid tissue and shaping Innate Lymphoid Cell development in the intestine [130,131]. Interestingly, mucosal IgG1 in mice targets *Akkermansia muciniphilia*, a bacteria that lives close to the intestinal epithelium and consumes mucus [132]. IgA also preferentially targets bacteria that live close to the epithelium, such as segmented filamentous bacteria [107]. Thus, perhaps both maternal milk-supplied IgG and IgA are a mechanism by which mothers can protect their children from invasion by the organisms that live closest to the intestine and are most likely to be capable of invasion. 

## 10. Conclusions

**Figure 1:**
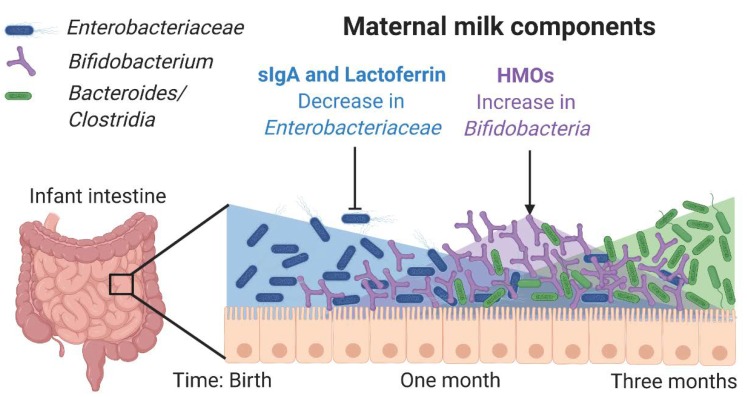
Maternal milk components shape the microbiota. *Enterobacteriaceae* is one of the first colonizers of the infant intestine and is controlled by secretory IgA (sIgA) from milk, prior to the infant’s own production of sIgA. Lactoferrin inhibits many types of *Enterobacteriaceae* by binding iron and preventing epithelial adhesion. Human milk oligosaccharides (HMOs) support the outgrowth of *Bifidobacteriaceae*, which can convert HMOs into Short Chain Fatty Acids, which are an important energy source for the intestinal epithelium and also contribute to immunoregulation. HMOs also serve as decoy receptors limiting bacterial association with the intestinal epithelium. Together these components induce an environment conducive to the colonization of the strict anaerobes that will compose the healthy adult intestinal microbiome.

The acquisition of microbiomes that are appropriate to the various body sites is an important component of development. If infants become colonized with microorganisms that are ill-suited to the barrier tissue site or are innately invasive and inflammatory, it can lead to both immediate and long-term health consequences. As a result, mammals have developed the ability to shape the intestinal microbiome of their infants and push it towards a state of health via the provision of milk (Figure 1). Here, we have reviewed the critical components of maternal milk that we know to shape the development of the microbiome, but important questions still remain. Unlike the anti-bacterial mechanisms of the internal organs, which are largely based on the killing of invasive organisms, the components at work in maternal milk are typically bacteriostatic and often we do not know how they mediate their effects to regulate the microbiome. For instance, over time the infant intestine becomes de-oxygenated, which is critical for the acquisition of fastidious anaerobes (*Bifidobacteria*, *Bacteroides*, *Clostridia*) necessary for the healthy function of the microbiome [133]. It has been hypothesized that facultative anaerobes can contribute to de-oxygenation, but these organisms are also dangerous for the host as this group (*Enterobacteriaceae*) contains many of the most inflammatory and invasive strains. How many facultative anaerobes are present in the infant intestine, how long they colonize, and how they associate to the infant intestine is clearly regulated by the components of mother’s milk, but how this might be balanced with a necessity for these organisms for de-oxygenation and colonization resistance is not known. For this and other questions related to maternal milk and the microbiome, we argue a comprehensive approach that tries to understand how particular host mediators affect bacterial colonization by shifts in bacterial gene expression is required. This ‘strain-centric’ approach is important because the effects of any given bioactive component of milk will change in response to shifts in the microbiome, driven either by new colonizers or evolution amongst long-term members. Furthermore, many bioactive factors, such as HMOs and the anti-bacterial repertoire of antibodies, differ significantly between mothers, implying that the fitness of any given bacterial isolate will be individualized to each host. Integrating analyses of the bacterial response to milk components with the host response, particularly the immune response, will lead to a better understanding of how the infant microbiome and thus maternal milk can shape the development of disease into adulthood.

## Figures and Tables

**Table 1:** Composition of term and preterm human milk.

Macronutrients	Term Milk	Preterm Milk
Protein (g/dL)	0.9	1.9–2.2
Fat (g/dL)	3.5	4.4–4.8
Carbohydrates (mainly lactose) (g/dL)	6.7	7.5
Energy (kcal/dL)	65 to 70	78

Table adapted from previous human milk studies [3,9] depicting the difference in macronutrient concentration in term and preterm milk.

**Table 2:** Proteins present in maternal milk.

Anti-Microbial Proteins	Nutritional Proteins
Lactoferrin	Bile-salt stimulated lipase
Secretory IgA, IgM, IgG	Haptocorrin
Kappa-casein	Folate binding protein
Lactoperoxidase	Alpha-lactalbumin
Haptocorrin	Casein
Lactadherin	

Table showing the different types of proteins in the maternal milk [7].

**Table 3:** Bioactive factors in the maternal milk.

Cells	Macrophages, Stem Cells, Bacteria
Anti-microbials	Immunoglobulins- Secretory IgA, IgM, IgG Lactoferrin, Lactadherin/MFG E8, Lysozyme, Complement C3, Antiviral mucins- MUC1, MUC4
Growth Factors	Epidermal growth factor (EGF), Nerve growth factor (NGF), Insulin-like growth factor (IGF), Transforming growth factor (TGF), taurine, polyamines, Heparin Binding EGF like growth factor (HB-EGF), Vascular Endothelial growth factor (VEGF), Erythropoietin
Cytokines, Chemokines and Anti-inflammatory factors	Tumor necrosis factor-alpha (TNF-α), Interferon-gamma (IFN-γ), Transforming growth factor-beta (TGF-β), prostaglandins, α_1_-antichymotrypsin, α_1_-antitrypsin, platelet-activating factor: acetyl hydrolase Interleukins- IL-6, IL-7, IL-8, IL-10 Chemokines- Granulocyte colony stimulating factor (G-CSF), Macrophage Migratory inhibitory factor (MIF)
Hormones	Calcitonin, Somatostatin, Adiponectin, Leptin, Ghrelin
Digestive enzymes	Amylase, Bile acid-stimulating esterase, Bile acid-stimulating lipases, Lipoprotein lipase
Transporters	Lactoferrin, Folate binder, Cobalamin binder, IGF binder, Thyroxine binder, Corticosteroid binder
Oligosaccharides and Glycans	Human Milk Oligosaccharides (HMOs), Gangliosides, Glycosaminoglycans

Adapted from previous studies explaining the importance of bioactive factors [9,64].
